# Lies, damned lies, and statistics

**DOI:** 10.1007/s40037-018-0425-x

**Published:** 2018-04-27

**Authors:** Geoff Norman

**Affiliations:** McMaster University, Hamilton, Canada

**Keywords:** Data analysis, Errors

## The story

Why do I do research? There are some obvious reasons. It pays well. I have lots of job security. I have lots of freedom to schedule my life. I have seen much of the world with other people footing the bill for airfare. And I get a thrill when the audience laughs at my jokes and applauds at the end.

But there is also an intrinsic reason. Good research is like writing your own detective story. Mother nature is the culprit and my goal is to get her to reveal her secrets. There is no greater thrill than the moment when you see your careful study design yield new insights about how things work, all at p less than 0.05; when, as Mosteller said so elegantly above, the data tell you an interesting story.

But it doesn’t always work out that way. All of us can describe studies that simply did not show the effects we were expecting. More likely than not, we expected a difference somewhere and did not find it. And I expect that most of the time, we never really know why.

Now that’s a good place for a small sidebar. At this point, many people would say that this is the time to publish it as a non-replication, and decry the fact of publication bias. While it is certainly the case that replication and lack of it is a serious issue in our field, I actually do not think that the present approach is that misguided. It seems to me that a certain sense of humility, assuming that whatever caused the lack of replication is probably something I did wrong, is likely an appropriate response. And certainly sometimes the failure to find what you are looking for actually leads to greater insights into the process you are studying.

On the other hand, things can and do go wrong as a result of errors that are uncovered. No one is immune to screw-ups. Certainly not me. I have had a number over the years spanning my whole career from my undergraduate physics days in Winnipeg to this summer, when two near debacles occurred. The early one involved some sloppiness in methodology, and I will discuss it only briefly. By contrast the two near catastrophes this year both involved a small, but devastating mistake in data reduction.

First, the distant past. My best friend and I were lab partners in a third year physics lab. The experiment we were to perform involved measuring the charge to mass ratio of an electron. It was really quite simple. We had a vacuum tube (those things that used to be inside huge radios that occupied every living room in Jimmy Stewart movies). In its simplest form it consisted of a vertical wire inside a metal cylinder, all enclosed in a light-bulb looking thing which was evacuated of air. When turned on, the wire glowed bright orange and emitted electrons. You put a constant voltage between the wire (cathode) and cylinder (anode) and the electrons flew from the wire to the cylinder, which showed up as an electric current. Now the sneaky bit. You put the whole thing inside a magnetic coil, so that as you ramped up the current on the coil, the magnetic field increased and the electrons started to curve. At some point they curved so much that they missed the cylinder, and the current dropped to zero. Knowing the voltages, currents, magnetic field and physical dimensions you could then work out the charge to mass ratio. Except that when we did it, the current dropped to zero then rose again and dropped again. We advanced all sorts of hypotheses about secondary ionization, etc. We even attracted the attention of a number of profs who came to examine our apparatus. For a brief period we were celebrities. We wrote it up, and prepared for glory.

Then Don Peterson took over. He replicated the results. Then he demonstrated that all he had to do was more carefully line the magnet up with the tube and it all went away. And so did our fame.

Rule No. 1: If something seems too good to be true, it probably is.

Rule out the obvious errors before you try to rewrite the universe. It was a lesson that stayed with me for a long time. A very long time. But not long enough it seems.

Flash forward to 2017. It was a very bad summer for screw-ups. Two different studies, in two very different areas, were completed. Both initially looked like complete washouts. But both suffered from a little tiny trivial mistake in data transformation that nearly ruined everything, at least until it was spotted. In the end, one study ended up verifying our fondest dreams—we defied Rule No. 1. The other did not, but along the way provided a rich landscape for real progress on our understanding.

Before I go into the gory details, a cautionary note. Because neither study is published yet, I must be deliberately vague about the details of design, hypotheses, etc. The study that ‘worked’ has yielded real insight into the research questions posed. So I cannot blow it by giving away all the secrets ahead of time. In due course, all will be revealed, but regrettably not at this time. The other one, even after we found the error, did not yield results that conformed to our expectations, but as a result did provide insights that enriched our understanding, and ultimately may lead to an entirely new research direction.

Let’s begin with the ‘every cloud has a silver lining’ study. The program began way back in 1999, when a medical student came to me with the idea to do a study of what was then called ‘CAI’ or Computer-Aided Instruction. We eventually settled on anatomy learning, and the possibility of dynamic presentations (as dynamic as you can get off an IBM 486, anyway).

We did it all on a computer screen. One group got to see the bones of the wrist, all in different colours, using posterior-anterior views; the other got to see the model rotate through a bunch of positions—a very primitive form of virtual reality. At test, we again put it on a computer screen, only this time the wrist was covered in skin and displayed at an oblique angle, and a pointer asked ‘What is this bone?’ The multiple views would do better, right? Wrong. They did worse and they did a lot worse when the participants had poor spatial ability [1]. Subsequent studies [2–4] again and again showed no advantage for virtual reality; on average it was as good as, but no better than, simple key views.

Then, in 2015, we did the comparison we had missed all this time. We compared virtual reality to key views to a plastic model [5]. We had three groups: As before, a key views group that got to observe the front, top, and side angles, and a virtual reality group, where you could look at any view or magnification under learner control, and a third plastic model group. The subject was the human pelvis, and the criterion outcome used a cadaver pelvis. To our astonishment, the plastic model won hands down. Typically a relative 50% advantage: 11/15 vs 7/15. As before, the virtual reality group performed exactly the same as the key views group.

But why did the plastic model have such a huge advantage? We went off to our cognitive psychologist colleague who said: ‘Well, it’s because they have much more sensory information’. But he was deliberately vague about just what that sensory information might be, so we were left to work out what might be causing the effects we had observed.

To cut a long story short, we tested about five different hypotheses. Each time we experimentally manipulated variables to examine what determined the presence or absence of the effect. For example, if we thought it was all to do with touch sensation, we could have tested them with thick gloves on or off (we didn’t, but you get the idea). Every time we did it made no difference, the plastic model continued to win hands down. Everything was highly significant. And across studies, the means were within a few percent of each other. Nothing we did made the effect go away. But while it gave us more confidence in our initial published finding, that meant we were no further ahead in understanding the superiority of the plastic model.

Then, through a combination of circumstances, we had a new idea that really appeared to hold promise. We did the experiment, and looked at the data with great anticipation as visions of Nobel Prizes danced in our heads.

Well, the effect we were looking for was there, sort of. But it was not significant. What was worse, nothing seemed to be coherent. The result from the previous study, where there was a large advantage for the physical model, was no longer there, even in the control condition that replicated the earlier published experiment.

Eventually, the students had to present the findings at a Research Day, so I had to come back to the data and do some further secondary analysis. Looking ahead to the ‘mother of all articles’ describing all of the manipulations, positive and negative, I decided to simply replicate the earlier robust findings so all the outputs would be in one place.

I started into it one Thursday morning. I had carefully put all the data from all the studies—a total of 10 groups of 20 learners each—into a single database. Critical to what will now unfold, the initial order of subjects (rows on the spreadsheet) was just as they came through the door, before they were assigned to a particular condition, and the study condition was described by a grouping variable. When I did the analysis, I transformed the data by sorting on this grouping variable, then I went ahead and did the analyses for particular groups.

To my complete horror, I could not replicate them. It made no sense. Nothing was significant. About an hour in I was suffering serious PTSD. Around 11, I became convinced that there was something wrong with the data, and faced the daunting task of sifting through 200 records to see where it went wrong. But when I started into it I realized that it was staring me in the face. While the grouping variable was re-sorted so all the subjects in a single group were together, all the remaining variables were in their original row. So the sorting was only on the grouping variable, and all this time I had been analyzing noise.

I quickly re-sorted things correctly. And now, when I did the analysis, all was revealed. All of the earlier results showing an advantage for the plastic model were replicated. Further, the critical test that explained why we got the effect emerged perfectly from the data.

What did we learn from this? The obvious lesson is to check and recheck every step of the way. Try analyses using different software. Check individual data records against the original forms. But a second lesson is that it is not good enough to just go looking for significant *p* values. The mistake was not uncovered because the *p* values were not significant. Rather, the data were not conforming to our expectations in direction and magnitude based on our own previous work. While that is not an ironclad guarantee of data problems, as the next example shows, it is one arrow in the quiver.

And now on to the second example, the ‘every cloud will rain on you anyway’ study.

I have been interested in clinical reasoning my entire career. In particular, I have been fascinated by the process of hypothesis generation, now called ‘System 1 thinking.’ The evidence suggests that this very rapid, unconscious process is based on prior exemplars, just like everyday categorization of cups, trees or butterflies [6]. The question we wanted to ask was what was the basis for retrieval—was it a single previous case (as we had shown in some studies)? Or does simply reminding people of the disease name do the trick [7]? Is it primarily based on objectively relevant cues? Or will any associations do it [8]?

We designed a devilishly clever study that had an initial ‘learning’ phase, where participants were asked to verify a clear case of Diagnosis A; then a test phase where they were asked to diagnose a new case that could be A or B. Within the learning phase we had four different kinds of prior cases. Everything was controlled and balanced. We did the study with emergency medicine specialists, residents and medical students, and it went off without a hitch. The data accumulated in a spreadsheet on our server, and then our research assistant created a summary Excel sheet that classified each case by type of prior case and level of learner.

I wanted to see a difference in both variables. It was a two-way ANOVA, looking at effect of Prior Case Condition (4 levels) and Education Level (3 levels). But it wasn’t there. There was no effect. And, the harder I looked, the more puzzling it became. Nothing worked. Experts looked like novices. No differences anywhere … nuttin’.

And then, poring over the code sheet like a Talmudic scholar, I found the answer. Turns out the ‘Condition’ variable in the spreadsheet that was generated was just essentially a descriptor for a label on a booklet of the materials. The difference between the prior cases was buried in a variable labelled ‘Verification’ (as in, ‘please verify these cases’).

So I reran it, with the right codes. Still nuttin’. And at that point, the research assistant and I had many sleepless nights as I railed about how it was coming out almost exactly the opposite of what we expected. She and I independently went back to the code sheets, and independently concluded that they were right, and our theory was wrong.

That’s where Mosteller comes in. Ma Nature was trying to tell us something really important. When I went back through a whole bunch of literature around exemplar theories it was staring me in the face. The effects or biases we were looking for only arise under particular conditions where the task is perceptual or ambiguous or induces lots of cognitive load. When the task is straightforward, participants will default to applying the rules, and the effects we anticipated will vanish. In our attempt to design the ultimate controlled experiment, we controlled away precisely the effects we were looking for. Of course, this amounts to no more than an untested hypothesis right now. But the larger story is that our materials created ‘boundary conditions’ that ultimately eliminated the effect we were looking for.

## The moral(s) of these happy and sad stories

1. It goes without saying that you have to be vigilant at all times. Check the data every time you go from one spreadsheet to another. Check individual data against the original coding forms. But all that is easier said than done.
2. A preoccupation with *p* values can lead to a blindness about what the data are telling you. It is important to graph the data, look at the means and so on. *P* values simply do not provide enough information to lead to really understanding the data.
3. Not every study simply reaffirms the theories in the literature. If it did, there would be little point in further research. But by embedding oneself in the literature prior to settling on a research question/design, you are better able to predict what the data should be doing. And you may also be better at understanding it when the data do not conform to your predictions, helping you to understand the literature at a deeper level.

## References

[1] Garg A, Norman GR, Spero L, Maheshwari P (1999). Do virtual computer models hinder anatomy learning?. Acad Med.

[2] Garg AX, Norman G, Spero L (2001). How medical students learn spatial anatomy. Lancet.

[3] Garg AX, Norman GR, Eva KW, Sperotable L, Sharan S (2002). Is there any real virtue of virtual reality?: the minor role of multiple orientations in learning anatomy from computers. Acad Med.

[4] Levinson AJ, Weaver B, Garside S, McGinn H, Norman GR (2007). Virtual reality and brain anatomy: a randomized trial of e‑learning instructional designs. Med Educ.

[5] Khot Z, Quinlan K, Norman GR, Wainman B (2013). The relative effectiveness of computer-based and traditional resources for education in anatomy. Anat Sci Educ.

[6] Norman G (2009). Dual processing and diagnostic errors. Adv Health Sci Educ Theory Pract.

[7] Schmidt HG, Mamede S, van den Berge K, van Gog T, van Saase JL, Rikers RM (2014). Exposure to media information about a disease can cause doctors to misdiagnose similar-looking clinical cases. Acad Med.

[8] Hatala R, Norman GR, Brooks LR (1999). Influence of a single example on subsequent electrocardiogram interpretation. Teach Learn Med.

